# Editorial: Molecular genetics and therapeutic advances in renal carcinoma

**DOI:** 10.3389/fonc.2022.1119373

**Published:** 2023-01-16

**Authors:** David M. Nanus, James H. Finke, Saby George

**Affiliations:** ^1^ Division of Hematology and Medical Oncology, Department of Medicine, Weill Cornell Medicine, New York, NY, United States; ^2^ Cleveland Clinic, Department of Immunology, Lerner Research Institute, Cleveland, OH, United States; ^3^ Department of Medicine, Roswell Park Cancer Institute, Buffalo, NY, United States

**Keywords:** renal cell carcinoma, long noncoding RNA, copper, iron, clear cell renal cell carcinoma, kidney cancer, molecular genetics

Over the past 30 years, genomic studies focusing predominantly of DNA alterations have led to a greater understanding of the genomic characteristics of various types of kidney cancers, including the multiple histologic subtypes. Clear cell renal cell carcinoma (ccRCC) remains the most common and most well studied kidney cancer, characterized by mutations in the Von Hippel-Lindau (VHL) gene resulting in expression of hypoxia inducible factors 1α and 2α (HIF-1α and HIF-2α). Numerous other genes are also commonly mutated in ccRCC including *PBRM1, SETD2, KDM5C, PTEN, BAP1, mTOR* and *TP53*, underscoring the importance of heterogeneity in different parts of the same tumor that may contribute to clinical progression (1, 2). Despite numerous molecular profiling studies describing the genomic landscapes of primary and metastatic ccRCC however, it is uncommon to identify a single alteration that suggests a targeted therapy should be prescribed to treat patients with treatment refractory ccRCC. Although immunotherapy has led to significant advances in our treatment of renal cancer patients, there clearly remains an unmet need to identify additional pathways that contribute to ccRCC initiation and progression.

This Frontiers special section on molecular genetics and therapeutic advances in renal carcinoma transitions from DNA genomic studies and explores the roles of long non-coding RNA (lncRNAs) in ccRCC. Zhang et al. reports an analysis of differentially expressed lncRNAs and mRNAs in plasma of ccRCC patients compared with healthy controls. Several differentially expressed lncRNAs and mRNAs were identified, which could conceivably be used as diagnostic or prognostic markers, although the biological function of these lncRNAs and mRNAs and their roles in ccRCC need to be assessed. Ju et al. performed a similar exploratory study, but this time identified differentially expressed lncRNAs in 54 pairs of ccRCC tissues compared with para-carcinoma normal tissue. They identified a number of lncRNAs highly expressed in ccRCCs and implicate LINC02747 as a potential regulator of renal cancer cell proliferation through adsorption of miR-608. Another approach taken by Zhong et al. examined the potential role lncRNAs on epithelial–mesenchymal transition (EMT) in renal cancers. They developed an EMT-related lncRNA risk signature and report that this lncRNA risk signature that can independently predict overall and disease-free survival in ccRCC patients. Two other manuscripts in this section study the role of RNAs in ccRCC. Chen et al. examined N6-methyladenosine (m6A) RNA methylation, a common RNA modification, in papillary RCC and developed a prognostic signature-based risk score; whereas Yu et al. performed single-cell RNA-seq on sporadic bilateral ccRCCs.

The role of the essential dietary metals copper and iron in RCC are also examined and reported on. Cuproptosis or copper-dependent controlled cell death has recently been found to be an important mediator of cell death in various malignancies (3). Ji et al. examines the expression of cuproptosis-related genes in 530 renal cancers, developing a cuproptosis score as a prognostic indicator. Greene et al. examine another metal, iron, reporting that that RCC has significantly higher iron staining scores compared with other solid cancers and, on average, >40 times higher than adjacent renal epithelium. Together, these novel studies suggest these essential dietary metals have an important but not yet well-defined role in RCC, and potentially there may be novel therapeutic strategies such as iron or copper depletion.

The final paper in this series is more clinical and provide an update the evolving role of adjuvant therapy for patients with high-risk relapse RCC (Renner et al.). Pembrolizumab was recently approved in the adjuvant space based on the Keynote-564 trial (4). Future studies should explore technologies like ctDNA for selecting patients with micro metastatic disease and thus high risk for recurrence, prior to adjuvant therapy. This series of papers provides the reader with a different scope to consider when one approaches the molecular alterations associated with renal cancers.

## Author contributions

All authors listed have made a substantial, direct, and intellectual contribution to the work and approved it for publication.

## References

[1] Turajlic S Xu H Litchfield K Rowan A Chambers T Lopez JI. Tracking cancer evolution reveals constrained routes to metastases: TRACERx renal. Cell (2018) 173(3):581–594.e12. doi: 10.1016/j.cell.2018.03.057 29656895 PMC5938365

[2] van der Mijn JC Eng KW Chandra P Fernandez E Ramazanoglu S Sigaras A. The genomic landscape of metastatic clear cell renal cell carcinoma after systemic therapy. Mol Oncol (2022) 16(12):2384–95. doi: 10.1002/1878-0261.13204 PMC9208073 35231161

[3] Chen L Min J Wang F. Copper homeostasis and cuproptosis in health and disease. Signal Transduct Target Ther (2022) 7(1):378. doi: 10.1038/s41392-022-01229-y 36414625 PMC9681860

[4] Choueiri TK Tomczak P Park SH Venugopal B Ferguson T Chang YH. Adjuvant pembrolizumab after nephrectomy in renal-cell carcinoma. N Engl J Med (2021) 385(8):683–94. doi: 10.1056/NEJMoa2106391 34407342

